# Ultrasound in managing extrapulmonary tuberculosis: a randomized controlled two-center study

**DOI:** 10.1186/s12879-020-05073-9

**Published:** 2020-05-15

**Authors:** Robert Ndege, Omary Ngome, Farida Bani, Yvan Temba, Herieth Wilson, Fiona Vanobberghen, Jerry Hella, Winfrid Gingo, Mohamed Sasamalo, Dorcas Mnzava, Namvua Kimera, Helen Hiza, John Wigayi, Herry Mapesi, Irene B. Kato, Francis Mhimbira, Klaus Reither, Manuel Battegay, Daniel H. Paris, Maja Weisser, Martin Rohacek

**Affiliations:** 1 Ifakara Health Institute, Ifakara, United Republic of Tanzania, Off Mlabani Passage, P. O Box 53, Ifakara, Tanzania; 2 St Francis Referral Hospital, Ifakara, United Republic of Tanzania; 3 Mwananyamala Regional Referral Hospital, Dar es salaam, United Republic of Tanzania; 4 Department of Medicine, Swiss Tropical and Public Health Institute, Basel, Switzerland; 5 Faculty of Medicine, University of Basel, Basel, Switzerland; 6 Division of Infectious Diseases, University Hospital Basel, Basel, Switzerland

**Keywords:** Sonography, FASH, Extrapulmonary, Tuberculosis, Sub-Saharan Africa

## Abstract

**Background:**

Patients with clinically suspected tuberculosis are often treated empirically, as diagnosis - especially of extrapulmonary tuberculosis - remains challenging. This leads to an overtreatment of tuberculosis and to underdiagnosis of possible differential diagnoses.

**Methods:**

This open-label, parallel-group, superiority randomized controlled trial is done in a rural and an urban center in Tanzania. HIV-positive and -negative adults (≥18 years) with clinically suspected extrapulmonary tuberculosis are randomized in a 1:1 ratio to an intervention- or control group, stratified by center and HIV status. The intervention consists of a management algorithm including extended focused assessment of sonography for HIV and tuberculosis (eFASH) in combination with chest X-ray and microbiological tests. Treatment with anti-tuberculosis drugs is started, if eFASH is positive, chest X-ray suggests tuberculosis, or a microbiological result is positive for tuberculosis. Patients in the control group are managed according national guidelines. Treatment is started if microbiology is positive or empirically according to the treating physician. The primary outcome is the proportion of correctly managed patients at 6 months (i.e patients who were treated with anti-tuberculosis treatment and had definite or probable tuberculosis, and patients who were not treated with anti-tuberculosis treatment and did not have tuberculosis). Secondary outcomes are the proportion of symptom-free patients at two and 6 months, and time to death. The sample size is 650 patients.

**Discussion:**

This study will determine, whether ultrasound in combination with other tests can increase the proportion of correctly managed patients with clinically suspected extrapulmonary tuberculosis, thus reducing overtreatment with anti-tuberculosis drugs.

**Trial registration:**

PACTR, Registration number: PACTR201712002829221, registered December 1st 2017.

## Background

In resource-limited settings with a high tuberculosis prevalence, almost half of patients are treated empirically with anti-tuberculosis drugs upon clinical suspicion only [1–3]. Besides lack of available tests, this is mainly due to the poor sensitivity of microbiological tests, especially in patients co-infected with human immunodeficiency virus (HIV) or in those with suspected extrapulmonary tuberculosis [4, 5]. The decision to treat tuberculosis based on clinical signs and symptoms only has a low accuracy for culture-confirmed tuberculosis, and 30–60% of these decisions lead to false treatment [1, 3, 6]. The consequence is, that patients are unnecessarily exposed to potentially toxic drugs for long time periods, and the diagnosis of their real disease such as other infections, cancer or heart failure is delayed, or missed. Additional diagnostic methods are therefore needed to distinguish tuberculosis from other diseases with similar symptoms.

Ultrasound is a widely available diagnostic tool, which is used in various medical fields. Focused Assessment with Sonography for HIV and Tuberculosis (FASH) is a useful tool to detect signs of extrapulmonary tuberculosis, namely pleural and pericardial effusion, enlarged abdominal lymph nodes, hypoechogenic lesions in the spleen and the liver, ascites and thickening of the bowel wall [7]. FASH can be performed as a point of care bedside test and can be taught to personnel with little or no previous experience in ultrasonography [8]. In our observational study from rural Tanzania including 191 HIV-positive and -negative patients with suspected pulmonary or extrapulmonary tuberculosis, abnormal chest X-ray, presence of ≥1 FASH-sign, and elevated body temperature were independently associated with confirmed tuberculosis. A combination of ≥1 FASH sign, abnormal chest X-ray, and temperature ≥ 37.5 °C had a high (99.1%) sensitivity but a low (35.2%) specificity for confirmed tuberculosis. We concluded that ultrasound might be useful to exclude tuberculosis, in combination with other tests [9]. In a recent Cochrane metanalysis including HIV-positive adults, FASH signs predicted tuberculosis with a moderate diagnostic performance only. The authors also concluded that ultrasound results should be considered in combination with other tests, such as chest X-ray and Xpert MTB/RIF® only [10]. No randomized controlled trial evaluating sonography for the diagnosis of tuberculosis has been done yet.

With our randomized controlled trial, we aim to determine whether sonography, added to chest X-ray and microbiological tests, has an impact on the proportion of correctly managed patients with suspected extrapulmonary tuberculosis, and on morbidity and mortality. We have added the sonography of axillary and cervical lymph nodes, of the chest, and of the vena cava to the FASH protocol, and call it extended FASH (eFASH). We hypothesize that the proportion of correctly managed patients after 6 months will be higher in the intervention group versus the control group.

## Methods

### Study design and setting

This is a two-center, open-label, parallel-group, superiority randomized controlled trial. Patients are consecutively recruited at St. Francis Referral Hospital, Ifakara and Mwananyamala Regional Referral Hospital, Dar es Salaam, United Republic of Tanzania.

St. Francis Referral Hospital serves a rural population of about 1 million people as a referral center. It includes the Chronic Diseases Clinic of Ifakara (CDCI), which cares for over 4000 people living with HIV [11, 12] and/or tuberculosis [13]. Mwananyamala Regional Referral Hospital serves an urban population of Dar es Salaam and is a referral center for tuberculosis within the Kinondoni district. The planned study period is 2 years, including 6 months of follow-up.

### Patients

All HIV-positive and -negative adults aged ≥18 years presenting to any department of the St. Francis Referral Hospital, CDCI or Mwananyamala Regional Referral Hospital are screened by trained study personnel. Eligible patients are those fulfilling clinical screening criteria for extrapulmonary tuberculosis, with or without concomitant signs of pulmonary tuberculosis (Table 1).

**Table 1:** **Eligibility criteria**

Inclusion criteria	- Adult (≥18 years) - Documented HIV status (HIV- positive and HIV- negative) - Suspected extrapulmonary tuberculosis*: presence of ≥1 sign or symptom of the following: • Fever of any duration, • Night sweat during 3 weeks within the last 4 weeks • Weight loss AND presence of ≥1 sign or symptom of the following: • Nuchal, cervical, axillary or generalized lymphadenopathy • Abdominal pain or ascites • Neurological symptoms (reduced consciousness, confusion, stiff neck, focal signs, persisting headache) • Presence of severe anaemia (hemoglobin < 8 g/dl) in an HIV infected patient under antiretroviral treatment • Local pain and spinal deformity, or arthritis suggesting spinal or osteoarticular tuberculosis • Painless hematuria or sterile pyuria, scrotal nodules or epididymal Hardening, or salpingitis suggesting urogenital tuberculosis • Chest x-ray with signs of miliary pattern, pleural effusion or suspected pericardial effusion AND No other obvious explanation for these signs * with or without concomitant signs of pulmonary tuberculosis (i.e cough or chest x-ray with upper lobe infiltrate, cavernous lesion)
Exclusion criteria	- Pregnancy
	- Patients already on anti-tuberculosis treatment
	- Refusal to participate or sign informed consent
	- Not available for follow-up visits

### Study procedures intervention group and control group

Before randomization, all eligible and consenting patients receive a clinical evaluation including an interview with a systematic assessment of symptoms. Physical examination including measuring body weight and height, blood pressure, heart rate, chest and abdominal examination, and palpation of lymph nodes is done by the study physician. All patients receive a chest X-ray, an HIV test if the HIV status is not documented and blood tests for hemoglobin, creatinine, and alanine aminotransferase. Microbiological analyses are performed as follows: If a patient can produce sputum, conventional Xpert MTB/RIF® (Cefeid, Sunnyvale, CA) is done. We decided to use the conventional Xpert in sputum because the Xpert MTB/RIF Ultra® assay has a lower specificity in sputum, and might generate false positive results [14]. Additionally, sputum samples are sent for solid culture after adding cetylpyridinium chloride and N-acetyl-l-cysteine-sodium hydroxide and are then inoculated on Löwenstein-Jensen medium [15]. For all patients, urine samples are collected and Xpert MTB/RIF Ultra® assay is used [16, 17].

**Fig. 1:**
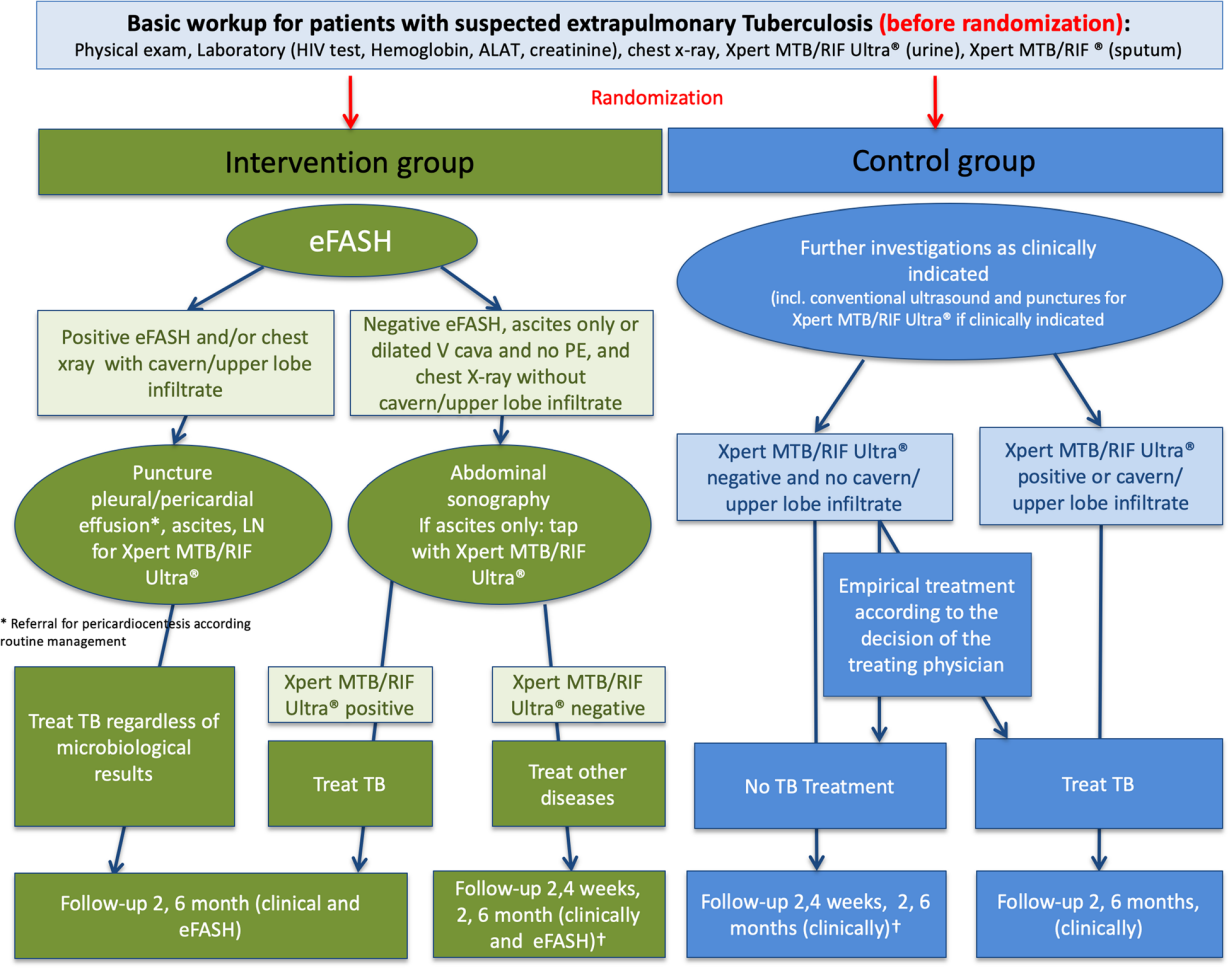
Algorithm for management of intervention group and control group. LN: lymph node; TB: Tuberculosis; eFASH: Extended Focused Assessment with Sonography for HIV and Tuberculosis; PE: pericardial effusion. **†** anti-tuberculosis treatment is started in case of a positive microbiological result

Patients allocated to the intervention group receive additionally an eFASH examination. Anti-tuberculosis management and treatment is done according to an algorithm (see Fig. 1). Patients allocated to the control group are treated according national guidelines [18]. eFASH consists of sonographic evaluation of the following:

- Original FASH signs: Pleural or pericardial effusion, hypoechogenic lesions in spleen and liver, abdominal lymphnodes > 1.5 cm, ascites, bowel wall thickening
- Extended signs: Diameter of the vena cava in order to detect signs for heart failure; axillary, nuchal and cervical lymph nodes > 1.5 cm; and B-lines and echogenic bright granular artefacts in the subpleural area in chest sonography. This is done by multiple scans of the main bilateral chest areas (anterior superior, lateral, posterior superior, posterior inferior of the left and right chest) [19].

eFASH is performed by two trained study doctors in patients in the intervention group only, before availability of microbiological results. All sonographical investigations are reviewed by an independent board-certified sonographer with a minimum experience of 5 years after the examination. This review will not influence the patient management, but it will be included in the final interpretation of the data. All sonographical examinations are done with a Sonobook 9 ultrasound machine (Chison, Jiangsu, China). In case of pleural fluid or ascites, fluid is taped and analysed using Xpert MTB/RIF Ultra® assay, liquid culture, and adenosine deaminase (ADA; Diazyme, Poway, USA). For liquid culture, bacteria growth indicator tube (MGIT), with a BACTEC 960 Instrument (BD Microbiology Systems, Sparks, MD) is used. In case of meningitis, lumbar puncture is performed and cerebrospinal fluid analysed with Xpert MTB/RIF Ultra® assay and liquid culture. In case of nuchal, cervical, or axillary lymphadenopathy, fine-needle aspiration biopsy (FNAB) is done for Ziehl-Neelsen stain, Giemsa- and Papanicolaou- stain for cytomorphology, and liquid culture. If liquid material is aspirated from a lymphnode, Xpert MTB/RIF Ultra® assay and culture is done. Patients with pericardial tamponade are referred for pericardiocentesis. Urine samples of 200 μl are frozen at − 80 °C in order to evaluate the novel lipoarabinomannan point of care test (FujiLAM) [20]. This will not affect the outcomes of this study.

In the intervention group, anti-tuberculosis treatment is started if any of the following is present: positive eFASH, chest x-ray with typical signs for tuberculosis, or positive microbiology from any site (Table 2). Anti-tuberculosis treatment is withheld and other causes than tuberculosis are considered if all microbiological tests are negative for *Mycobacterium tuberculosis*; eFASH does not show signs for extrapulmonary tuberculosis and no cavernous lesion, upper-lobe infiltrate or miliary pattern on the chest x-ray are seen. If the vena cava is dilated > 2 cm and collapsing less than 50% during inspiration and no eFASH signs are present, heart failure is considered. Presence of ascites with no other eFASH signs points at pelvic inflammatory disease (PID) in females, liver cirrhosis, nephrotic syndrome, heart failure, or at malignancy. Thickened ileum wall with loss of wall architecture and no other eFASH signs points at bacterial ileitis or Morbus Crohn. In case of ascites with otherwise negative eFASH, a full abdominal sonography to look for these diseases is done (Fig. 1).

**Table 2:** Criteria for starting anti-tuberculous treatment in the intervention group

Positive eFASH	Multiple hypoechogenic lesions in the spleen or liver
	Pericardial effusion and no other clinical explanation
	Pleural effusion and no clinical or sonographic sign for heart failure (i.e normal V cava)
	Subpleural echogenic granular artefacts and B-lines
	abdominal, axillary, nuchal or cervical lymphnodes > 1.5 cm and no other clinical explanation for it
	Thickened ileum wall (> 4 mm) and loss of wall architecture and at least one another eFASH sign
	Ascites and at least one another eFASH sign
Chest X-ray with typical signs for tuberculosis	Cavernous lesion, upper-lobe infiltrate or miliary pattern
Positive microbiological result from any site	Positive Xpert MTB/RIF® assay and/or culture in sputum, pleural fluid, ascites, cerebrospinal fluid, or urine;
	Adenosine deaminase (ADA) ≥40 U/ml in pleural fluid, [21], ADA ≥ 35 U/ml in ascetic fluid [22], or ADA ≥35 U/ml in pericardial fluid [5];
	Positive fine needle aspiration result of lymph nodes (Xpert MTB/RIF® assay, culture, cytomorphology, identification of acid fast bacilli)

Patients allocated to the control group are diagnosed according to national guidelines [18] and the decision of the treating physician. Patients receive empirical treatment according to the discretion of the treating physician (Fig. 1). The treating physician of a control group patient is allowed to use ultrasound for puncturing pleural -or pericardial effusion, ascites, or lymph nodes, or in case of a serious acute problem during follow-up (e.g. suspected urolithiasis, cholecystitis, intestinal obstruction, perforation, appendicitis, or ruptured ectopic pregnancy). All patients who receive anti-TB treatment are routinely supervised by the treating Tuberculosis clinics of both sites by drug tablet return checking (weekly visits as per governmental guidelines, which can be also in a peripheral dispensary).

### Follow-up

In both groups, follow-up visits take place at two and 6 months after enrollment. Follow up visits include physical examination, and eFASH in the intervention group. If anti-tuberculosis treatment is not administered at enrollment, additional follow-up visits are done at two and 4 weeks. Transportation costs for study visits is paid by the study budget. If a patient misses an appointment, he or she will receive a phone call from the study team. If a patient is not reachable by phone, he or she is sought by an established collaboration with community health care workers or by physical tracking to the patients home by motorbike.

### Outcomes

The primary outcome is the proportion of correctly managed patients at 6 months. Secondary outcomes are the proportion of patients without symptoms at two and 6 months, and time to death. Other outcomes of interest are the proportions of patients with microbiologically-proven tuberculosis; with probable tuberculosis; with no tuberculosis; with a diagnosis other than tuberculosis; and receiving an invasive procedure. Safety outcomes are the occurrence of pneumothorax, major bleeding, and post-expansion pulmonary edema after interventions such as pleural tapping, ascites puncture or fine-needle aspiration.

### Definitions

A patient is defined as having definite tuberculosis if any of the microbiological tests is positive (Xpert TB/RIF®, Xpert Ultra TB/RIF®, culture, acid fast bacilli in fine-needle aspiration from lymphnodes, adenosine deaminase (ADA) ≥ 40 U/ml in pleural fluid [21], ADA ≥ 35 U/ml in ascetic fluid [22], or ADA ≥ 35 U/ml in pericardial fluid [5].

A patient is defined as having probable tuberculosis if all microbiological tests are negative, and clinical, radiological and sonographic (in the intervention group) signs improve, and body weight is stable or increases after 2 months of anti-tuberculosis treatment. A patient is defined not to have tuberculosis if all microbiological tests were negative, and (a) clinical signs, symptoms and radiological signs (and in patients in the intervention group sonographic signs) improved, and body weight increased, without start of anti-tuberculosis treatment or (b) a diagnosis other than tuberculosis was found explaining the symptoms in a patient with or without anti-tuberculosis treatment.

A correctly managed patient is defined as one who had definite or probable tuberculosis and was treated with anti-tuberculosis treatment; or a patient who did not have tuberculosis and was not treated with anti-tuberculosis treatment.

### Timeline

Recruitment started September 23rd 2018 and was initially planned until the end of February 2020 with a follow-up until August 2020 (Table S1). Due to initial slow recruitment, we planned a prolongation of recruitment until June 2020 and of follow-up until December 2020. However, due to the SARS-CoV-2 pandemic, recruitment had to be paused on March 30th 2020, based on the recommendation of the National Institute for Medical Research, Tanzania. Until March 30th 2020, a total of 538 patients were included. To date, it is not possible to determine the date when recruitment will be resumed. Therefore, the study period will be prolonged again by the time that recruitment was paused.

### Recruitment

Patients are recruited in the emergency department, the outpatient clinics, and the wards of the St Francis Referral Hospital, Ifakara, and at the Chronic Disease Clinic Ifakara, and at the wards and outpatient clinics of the Mwanyamamala Regional Hospital, Dar es salaam, were tuberculosis patients were recruited for several other studies. All medical staff is regularly informed about the study, and posters with inclusion criteria and the phone number of the study team were distributed.

### Randomization

The randomization list was prepared in advance by the statistician, stratified by site (Ifakara and Mwananyamala) and HIV status (positive or negative) with randomly-varying block sizes. Patient allocation was done in a 1:1 ratio to the intervention or control group.

The sequence was concealed by using envelopes prepared by an independent person, based on the list prepared by the statistician. The envelopes are opaque and sealed, labelled only with the stratification information and a sequential number. Inside the envelope is a piece of paper confirming the stratification information and sequential number, and indicating the group allocation. The envelopes are stored securely. Checks are performed intermittently during enrolment to ensure that the randomization sequence is being respected.

Allocation of patients to the intervention or control group is done by the study clinical officers at each study site, after history taking, physical examination, collection of blood, urine and sputum for laboratory and microbiological examinations, chest X-ray, HIV testing, and informed consent. Based on the stratification of the patients (i.e site and HIV status), the study clinical officers take the next sequentially numbered envelope. They verify the information inside the envelope, and store the allocation paper in the patient file for source document verification if necessary.

### Sample size

Assumptions for the sample size calculation were based on data from the literature [1, 3, 6] and preliminary analysis of our observational study [9] (Table S2). Based on these assumptions, 73 and 85% of patients in the control and intervention groups, respectively, would be correctly managed. For a power of 95% with a two-sided alpha of 0.05, this yields a sample size of 592 patients. Assuming a lost to follow-up of 10%, 650 patients are required.

### Data collection and management

All data are managed using Epidata (http://www.epidata.dk/). Data are stored on a local computer. Data extraction is done daily and stored on a secured cloud storage service accessible only by study team members through a user name-password authentication. Since clinical, microbiological and sonographic findings are crucial for the decision to treat or not to treat a patient according to the protocol, these findings are accessible to the members of the study group. Information about results of microbiological data are given to the treating physicians of the patients in the control group. Direct access to source documents is permitted for purposes of monitoring and inspections. Only investigators and the monitoring team have access to the dataset. All data are checked and verified by the responsible investigators.

### Statistical analyses

Analyses and reporting will follow CONSORT guidelines (http://www.consort-statement.org/consort-2010) following intention to treat principles. Enrolment, randomisation and follow-up will be described using a flowchart. Baseline characteristics and outcomes will be described by group using frequencies and percentages for categorical variables and medians and interquartile ranges for continuous outcomes. The primary outcome will be compared between groups using logistic regression, reporting odds ratios with 95% confidence intervals. We will adjust for the stratification variables [23], and in sensitivity analyses for other baseline factors with imbalances between groups (by visual inspection, with no formal testing performed across groups) [24] or associated with missing primary outcome data [25]. We will assess effect modification of the primary outcome separately by HIV status and site, by using similar methods as for the primary outcome and an interaction between group and HIV status or site, respectively. Analyses of the categorical secondary and safety outcomes will follow similar methods as for the primary outcome, and with proportional hazards models for the time to death outcome if there are sufficient numbers of events. Inter-rater reliability of eFASH findings between the sonographer and the independent reviewer will be evaluated. A full statistical analysis plan will be developed.

### Monitoring and auditing

Monitoring is done by independent members of the Ifakara Health Institute. Monitoring is done at the beginning of the study, after 1 year, and at the end of the study. Health hazard of trial interventions that require measures are reported to the sponsor-investigator, to the study monitoring board, and to the local ethics committee. No interim analysis requiring a data monitoring committee is planned.

## Discussion

In 2018 an estimated 10 million new tuberculosis cases occurred worldwide, and 1.45 million people died of the disease [26]. Clinical symptoms can be sensitive, but not specific for tuberculosis [27]. Moreover, in HIV positive people on antiretroviral therapy, the absence of cough, fever, night sweats, or weight loss does not exclude tuberculosis, because of the low sensitivity of these symptoms in this group of patients [28]. Microbiological testing as the gold-standard remains challenging, with severely ill patients and patients with extrapulmonary tuberculosis who are often unable to expectorate sputum. Invasive sampling to acquire material for microbiology is mostly not available [29]. Furthermore, a strategy relying on spontaneously expectorated sputum is inadequate in about one third of tuberculosis cases [30]. Moreover, patients with tuberculosis and HIV often have low bacillary loads in sputum and other body cavity fluids, which reduces further the sensitivity of culture and Xpert MTB/RIF® assay [31]. Therefore, patients are often empirically treated despite negative microbiological tests [6]. Overtreatment of patients who do not have tuberculosis generates unnecessary costs, exposes patients to potentially toxic drugs, and might delay the diagnosis of the real cause of the disease. To our knowledge, this is the first randomized controlled trial to assess whether sonography, combined with chest X-ray and established microbiological tests, can increase the proportion of correctly managed patients with clinically suspected extrapulmonary tuberculosis.

This trial has some limitations: First, we cannot prevent doctors of patients in the control group from using sonography as part of routine care (not according eFASH protocol), which might reduce the real difference in outcomes between the groups. Second, the study is not blinded. However, results will reflect real life scenarios. Third, according to our experience in the observational study [9], loss to follow-up is a frequent challenge especially in the rural setting. We have attempted to address this through increasing our sample size to allow for some loss to follow-up. Fourth, this study is done in an environment with high tuberculosis prevalence, and findings cannot be transferred to low prevalence settings.

In conclusion, this study is the first randomized controlled trial investigating ultrasound in the management of extrapulmonary tuberculosis. We hypothesize that ultrasound, added to other tests, will increase the proportion of correctly-managed patients, and therefore reduce overtreatment with anti-tuberculosis drugs.

## Supplementary information

**Additional file 1: Table S1.** Study Schedule and Milestones. *Dates as planned initially. **Table S2.** Assumptions for sample size calculation. Shaded cells show those with correct management. TB=tuberculosis. *Based on the treatment decision made within the first 48 hours after primary evaluation.

## Data Availability

Data and Materials are available on justified request. Please contact the corresponding author Dr. Martin Rohacek if data are requested.

## Abbreviations

ADA: Adenosine deaminase
CDC: Chronic Diseases Clinic of Ifakara
eFASH: Extended focused assessment of sonography for HIV and tuberculosis
FASH: Focused assessment of sonography for HIV and tuberculosis
HIV: Human immunodeficiency virus
LN: Lymph node
TB: Tuberculosis
PE: Pericardial effusion
