# Supplementary material for: Ultrasound in managing extrapulmonary tuberculosis: a randomized controlled two-center study
Source: BMC Infect Dis. 2020 May 15;20:349. doi: 10.1186/s12879-020-05073-9 (PMC7226714; doi:10.1186/s12879-020-05073-9)
Supplement: Supplementary file 1 — Additional file 1: Table S1. Study Schedule and Milestones. *Dates as planned initially. Table S2. Assumptions for sample size calculation. Shaded cells show those with correct management. TB=tuberculosis. *Based on the treatment decision made within the first 48 hours after primary evaluation. [file 12879_2020_5073_MOESM1_ESM.docx]

**Table S1: Study Schedule and Milestones**

| STUDY PERIOD | Pre-Allocation | | Post-Allocation | | |
| --- | --- | --- | --- | --- | --- |
|  | Enrolment | Allocation | Baseline | Follow-Up | Close-out |
| TIME POINT | 0 | 0 | 0 | 2 months | 6 months |
| ENROLMENT | x |  |  |  |  |
| Eligibility screen | x |  |  |  |  |
| Informed consent | x |  |  |  |  |
| Allocation |  | x |  |  |  |
| INTERVENTION (eFASH) |  |  |  |  |  |
| Intervention Group |  |  | x | x | x |
| Control Group |  |  |  |  |  |
| ASSESSMENTS |  |  |  |  |  |
| Baseline variables: |  |  |  |  |  |
| Age | x |  |  |  |  |
| sex | x |  |  |  |  |
| HIV-status | x |  |  |  |  |
| Medical history/symptoms | x |  |  | x | x |
| Physical examination | x |  |  | x | x |
| Blood Tests | x |  |  | (x) | (x) |
| Chest x-ray | x |  |  | (x) | (x) |
| XpertMTB/RIF urine, sputum | x |  |  |  |  |
| Management and medication plan |  |  | x | x | x |
| Outcome variables: |  |  |  |  |  |
| Correct management |  |  |  |  | x |
| Freedom of symptoms |  |  |  | x | x |
| Death |  |  |  |  | x |
| Safety outcomes |  |  | x | x | x |

| *9/2017-8/2018** | Study year 1: September 2018-August 2019* | | Study year 2: September 2019-August 2020* | |
| --- | --- | --- | --- | --- |
| **Milestones:**  _Study protocol finalized  _Contracts/agreements signed  _Required approvals and authorizations (Ethikkommittee der Nord-und Zentralschweiz, IHI-RB Tanzania, NIMR, Tanzania)  _Data management system ready  _QA/monitoring system ready  _Safety reporting system ready  _Study team trained | **Milestones**:  1.9.2018*: First patient first visit,  both study sites active | | **Milestones:**  31.02.2020*: Last patient included  31.8.2020*: Last follow-up,  Database cleaned, Final analysis of endpoints | |
|  | Number of patients included after 6 months: 217 |  | Number of patients included after 18 months: 650 |  |
|  | Number of patients included after 12 months: 433 |  |  | |

*Dates as planned initially

**Table S2: Assumptions for sample size calculation**

|  |  | **Final diagnosis at 6 months** | | | **Correct management** |
| --- | --- | --- | --- | --- | --- |
|  |  | **Definite TB** | **Probable TB** | **No TB** |  |
| **Presumptive diagnosis at baseline** | **Control group** |  |  |  |  |
|  | Definite TB (and therefore treated*) | 35% | 0 | 0 | 35% |
|  | Probable TB (and therefore treated*) | 0 | 30% | 25% | 30% |
|  | No TB (and therefore not treated*) | 0 | 2% | 8% | 8% |
|  | Total |  |  |  | **73%** |
|  | **Intervention group** |  |  |  |  |
|  | Definite TB (and therefore treated*) | 35% | 0 | 0 | 35% |
|  | Probable TB (and therefore treated*) | 0 | 25% | 10% | 25% |
|  | No TB (and therefore not treated*) | 0 | 5% | 25% | 25% |
|  | Total |  |  |  | **85%** |

Shaded cells show those with correct management. TB=tuberculosis. *Based on the treatment decision made within the first 48 hours after primary evaluation
